# Predicting Dementia Risk: Progress, Pitfalls, and Priorities

**DOI:** 10.3389/ijph.2026.1609520

**Published:** 2026-01-22

**Authors:** Patricia Nistor, Osvaldo Espin-Garcia, Zul Merali, Shehzad Ali

**Affiliations:** 1 Department of Epidemiology and Biostatistics, Western University, London, ON, Canada; 2 Department of Biostatistics, University Health Network, Toronto, ON, Canada; 3 Dalla Lana School of Public Health and Department of Statistical Sciences, University of Toronto, Toronto, ON, Canada; 4 Brain and Mind Institute, The Aga Khan University - Kenya, Nairobi, ON, Kenya; 5 Department of Health Sciences, University of York, York, ON, United Kingdom; 6 Institute for Mental Health Policy Research, Centre for Addiction and Mental Health, Toronto, Canada; 7 Department of Psychology, Macquarie University, Sydney, NSW, Australia; 8 WHO Collaborating Centre for Knowledge Translation and Health Technology Assessment in Health Equity, Ottawa, ON, Canada

**Keywords:** Dementia, Alzheimer’s disease, aging population, risk prediction, algorithms

## Introduction

As populations age, identifying individuals at high risk for dementia has become a cornerstone of prevention-oriented public health policy. Accurate and scalable risk stratification could enable early intervention and resource targeting. However, existing models face methodological limitations, including constraints in available data and follow-up duration, challenges in dementia ascertainment, heterogeneity in modelling approaches, and limited external validation. This commentary discusses these challenges and identifies methodological priorities for developing dementia risk algorithms that are robust and suitable for real-world implementation.

## Current Challenges of Dementia Risk Prediction

### Data Limitations

Existing longitudinal datasets exhibit significant limitations in diversity and temporal scope. Most prediction models use cohorts from high-income countries which constrains generalizability. Moreover, most datasets, except for a few multi-decade cohorts such as the Whitehall II study, have follow-up periods that are too short to capture life-course risk trajectories [1].

These limitations are further compounded by heterogeneity in study designs [2] which hampers comparisons across cohorts and algorithms. Selection bias further compromises representativeness as volunteer-based cohorts may underrepresent vulnerable populations with limited healthcare access, raising concerns about the generalizability of these algorithms across diverse populations and healthcare settings.

### Outcome Ascertainment Heterogeneity

The establishment of dementia diagnosis is a necessary step for risk prediction; however, dementia ascertainment varies substantially across contexts. Community-based diagnoses captured through billing codes in administrative data often occur at advanced stages of disease, reflecting delayed provider recognition and variability in access to healthcare [3]. Research cohorts, such as the UK Biobank, employ regular cognitive assessments to identify cases, and may detect dementia early. However, they rely on participant-reported diagnoses between assessment waves, potentially introducing recall bias and misclassification [2, 4]. More in-depth ascertainment of dementia status, such as interviews conducted by trained research personnel [2, 4], may not be feasible in non-specialist clinical settings. In addition, even within similar datasets, investigators employ varying outcome definitions, from composite cognitive measures to single diagnostic codes, limiting direct comparison of model performance [1, 5].

### Methodological Heterogeneity

Current algorithm development exhibits substantial methodological diversity. Most studies conduct only internal validation through sample splitting [4], with unstandardized partitioning procedures and performance metrics. Additionally, methods used to develop risk stratification algorithms vary, reflecting the heterogeneity across data sources.

Reporting remains inconsistent; many studies provide insufficient detail for replication, and code availability remains exceptional rather than standard. Furthermore, many studies do not include sufficient detail for replication or evaluation of the cut-off values used by the algorithms to classify individuals into different risk levels. This opacity prevents independent validation and methodological refinement.

Few studies systematically evaluate incremental predictive value from biomarkers *versus* lifestyle and clinical variables, limiting evidence-based variable selection. Measures of predictive performance also vary, with common methods including Area Under the Receiver Operating Characteristic curve [4] and further subgroup and sensitivity analyses being conducted [5]. Model external validation and calibration across diverse populations receives minimal attention despite being essential for clinical implementation.

### Limited Clinical Applicability

Algorithms incorporating neuroimaging, cerebrospinal fluid markers, or genetic data demonstrate superior discrimination but remain infeasible for primary care implementation [6]. With the rise of large datasets and high dimensional data, comes an increase in the use of machine learning models for predicting risk [7], in some cases outperforming traditional modelling techniques, like logistic regression [4]. However, due to practical constraints in primary care [8], implementation of such complex models can be difficult. Conversely, parsimonious models using readily available variables often sacrifice substantial predictive performance. Evidence on the performance–feasibility trade-off across different healthcare contexts remains limited. Such evaluation could meaningfully inform decision-makers and clinicians about the systematic integration of these algorithms.

## Recommendations for Advancement

### Data Infrastructure Enhancement

While continued refinement of dementia risk prediction models remains important, further progress will increasingly depend on addressing limitations in the underlying data infrastructure. Coordinated multi-national datasets, spanning a wider range of populations and healthcare systems, could strengthen external validity by enabling model development and evaluation beyond a small set of well-studied cohort [9]. Explicit inclusion of low- and middle-income countries is particularly important, both to improve global generalizability and to avoid perpetuating evidence gaps driven by data availability rather than epidemiology. Complementary linkage of administrative health data with research cohorts offers a pragmatic means of improving population representativeness while incorporating longitudinal health services and utilization information at scale. Extending such linkages across childhood, midlife, and later life would allow risk trajectories to be examined over time horizons that remain inaccessible in most existing studies. This is critically important because the biological underpinnings often develop over years, prior to symptomatic manifestation. To maximize their value, such data initiatives should adopt open or minimum-barrier access models, enabling broader participation in model development and external validation while reducing duplication and fragmentation across research efforts [10].

### Outcome Standardization

Implementing consensus diagnostic criteria across studies would improve comparability. When using administrative data, algorithms should incorporate diagnostic delay adjustments through back-calculation or validation against research diagnoses. Self-reported dementia outcomes require verification protocols through cognitive batteries or checking previous medical and/or administrative records [8]. This is of particular concern as individuals may potentially forget to report their diagnosis because of symptoms of their diagnosis [8], especially in studies with long follow-up intervals. Transparent reporting of outcome definitions – including specific codes, thresholds, and composite variable construction – should become mandatory.

### Methodological Rigor

Establishing reporting guidelines specific to dementia risk algorithms would promote standardization. Required elements should include: detailed splitting procedures, cross-validation approaches, missing data handling, and subgroup performance metrics. Public code repositories should become standard, enabling replication and refinement. Systematic evaluation of variable importance and incremental predictive value across predictor categories would inform optimal model complexity for different settings.

Studies should consider moving beyond discrimination metrics to assess calibration, reclassification, and clinical utility measures. External validation in diverse populations should become standard before clinical translation. Understanding how these models perform in different populations would provide valuable evidence for which algorithms to implement into which settings to best serve each specific population and sub-population.

### Clinical Translation

Improving translation from research into clinical care is key, particularly for diseases of older age [10]. Thus, developing context-specific algorithms rather than universal models may optimize real-world performance. Systematic evaluation of performance-complexity trade-offs should identify minimal variable sets maintaining acceptable accuracy. Primary care-focused models utilizing readily available variables, that can seamlessly integrate into complex primary care workflows, warrant priority. These could include non-invasive blood markers that can be derived from routine clinical blood work, for example, cholesterol levels [4]. Effective implementation should include these accurate, simplified models, complementing physician knowledge and possible additional training in diseases of older age [10].

### Conclusion

Dementia risk prediction is limited less by the availability of modelling techniques than by the data and evaluative frameworks on which these techniques depend. Algorithms developed in narrowly defined cohorts, assessed using heterogeneous outcome definitions, and reported with limited transparency may demonstrate good apparent performance yet may fail to generalize across populations, healthcare systems, and care settings. Addressing these constraints requires a shift in emphasis toward data infrastructures that improve representativeness and longitudinal coverage, including explicit inclusion of data from low- and middle-income countries; outcome definitions that are sufficiently standardized to permit meaningful comparison and validation; and methodological norms that prioritize reproducibility, calibration, and external validation. Without such changes, advances in model development are unlikely to translate into reliable, equitable, or scalable tools for dementia detection and prevention. Progress in this field therefore depends on aligning methodological innovation with data quality, transparency, equity and real-world applicability as central objectives.
